# Effects of feedback literacy scripts and a second-rater mechanism on EAP writing revision in generative AI-supported contexts

**DOI:** 10.3389/fpsyg.2026.1835593

**Published:** 2026-06-11

**Authors:** Xiao-Chao Yao

**Affiliations:** Foreign Language Teaching Department, Hainan Vocational University of Science and Technology, Haikou, Hainan, China

**Keywords:** classroom simulation, EAP writing, feedback literacy, feedback uptake, generative artificial intelligence

## Abstract

**Introduction:**

As generative artificial intelligence increasingly enters university English for Academic Purposes (EAP) writing instruction, a key question is how AI-generated feedback can be translated into effective revision. Existing studies have mainly examined generative AI as a tool for feedback delivery, writing support, and instructional efficiency, whereas the links among feedback uptake, revision depth, and delayed retention remain insufficiently explored.

**Methods:**

To address this gap, this study developed a large language model–driven classroom simulation framework and adopted an exploratory 2 × 2 design. Two treatment variables were examined: whether a feedback literacy script was provided and whether an AI second-review mechanism was introduced. The study simulated 100 B2-level writers across different experimental conditions and tracked writing scores, effective feedback uptake, revision depth, and retention performance across Task 1, Task 2, and delayed Task 3.

**Results:**

The results revealed four main patterns. First, the feedback literacy script produced a clear positive effect during the initial revision stage. In Task 1, the scripted group gained 5.75 more points on average than the non-scripted group, and the regression coefficient was 7.09, with an exact permutation test yielding *p* = 0.0286. Second, this effect weakened in Task 2. Third, the script did not substantially increase effective feedback uptake, but it redirected revision toward the argument level; argument-level revision was positively associated with score gain (r = 0.755, *p* = 0.0304). Fourth, the second-review mechanism did not produce a stable benefit.

**Discussion:**

Overall, the findings suggest that improvement in AI-supported EAP revision depends less on the addition of more feedback sources than on learners’ ability to process feedback through structured decision-making. Feedback literacy scripts may therefore be more useful as cognitive and procedural scaffolds for revision than as simple prompts for adopting AI-generated feedback.

## 1 Introduction

With the growing use of generative AI in university writing classrooms, the key question for EAP instructors is no longer whether students should use AI, but how AI-generated feedback can be turned into actual learning. Recent discussions of ChatGPT in higher education have moved between two views. On the one hand, researchers highlight its efficiency, immediacy, and accessibility, arguing that it can support language use, text organization, and argument development. On the other hand, increasing attention has been paid to its possible risks, including weaker independent judgment, blurred authorial responsibility, and challenges to traditional writing assessment (Baidoo-Anu and Ansah, 2023; Grassini, 2023; Kasneci et al., 2023). This tension is particularly strong in EAP writing, where students must manage stance, evidence, organization, register, and accuracy at the same time. When AI can instantly generate multiple plausible suggestions, students may gain not stronger writing ability, but a convenient substitute for judgment.

This issue is closely related to a long-standing concern in written feedback research. Early debates over corrective feedback were not simply about whether teachers should give comments, but whether those comments could be noticed, understood, evaluated, and turned into writing development. Truscott’s (1996) critique of grammar correction, Ferris’s (1999) response, and later work by Hyland and Hyland (2006), Ellis (2008), Ferris (2010), and Bitchener and Ferris (2012) all suggest that the value of feedback depends less on the existence of suggestions than on how learners process them (Truscott, 1996; Ferris, 1999, 2010; Hyland and Hyland, 2006; Ellis, 2008; Bitchener and Ferris, 2012). The same feedback may be adopted immediately but lead only to surface-level change, or it may be ignored at first but later contribute to a deeper reorganization of the text. Research on uptake, noticing, and engagement has repeatedly shown that feedback does not move directly from input to outcome, but involves judgment, selection, and risk-taking (Han and Hyland, 2015).

The rise of automated writing evaluation and automated feedback systems extended this issue from teacher feedback to machine feedback. Warschauer and Grimes (2008), Shermis and Burstein (2013), and Cotos (2014) showed that the value of machine feedback lies not in replacing teachers, but in changing the speed, frequency, and accessibility of feedback (Warschauer and Grimes, 2008; Shermis and Burstein, 2013; Cotos, 2014). With generative AI, however, greater technical power has not removed the cognitive difficulty of processing feedback. Studies by Wilson and Czik (2016), Koltovskaia (2020), Ranalli (2021), and Huawei and Aryadoust (2023) show that learners respond to automated feedback in different ways: some treat it as useful guidance, some remain uncertain about its credibility, and some recognize problems but do not know how to prioritize revisions (Wilson and Czik, 2016; Koltovskaia, 2020; Huawei and Aryadoust, 2023). Generative AI has made this issue even more visible. Although large language models can produce coherent and wide-ranging feedback with clear potential for writing support (Mizumoto and Eguchi, 2023; Guo and Wang, 2024), research also suggests that students in multi-source feedback environments often struggle to compare, filter, and prioritize suggestions (Yan and Zhang, 2024). For EAP teachers, the main issue is therefore no longer how to obtain more feedback, but how to help students process feedback effectively.

Against this background, feedback literacy offers a useful lens for understanding AI-supported writing. Carless and Boud (2018) define student feedback literacy as the capacity to understand, evaluate, manage, and use feedback information, while Winstone et al. (2017) describe feedback reception and enactment as a series of observable learning processes. Recent studies in L2 writing have further shown that clearer procedures for processing feedback can strengthen learners’ engagement and revision planning (Winstone et al., 2017; Carless and Boud, 2018). More recently, Liu and Deris (2025) and Shu (2025) have proposed the idea of AI feedback literacy, emphasizing learners’ ability to interpret, filter, and anticipate the value of AI-generated feedback (Liu and Deris, 2025; Shu, 2025). However, research still pays more attention to perceptions and one-time outcomes than to the links among feedback scripts, uptake behavior, revision depth, and writing performance. It also remains unclear whether adding a second-review mechanism improves feedback use or simply increases learners’ processing burden. The present study addresses this issue from a mechanism-oriented perspective. It starts from the proposition that AI-generated feedback contributes to writing improvement only when learners can interpret, prioritize, and enact suggestions during revision. On this view, feedback literacy scripts and a second-review mechanism matter because they shape feedback processing and the depth at which revision is carried out. Using an exploratory 2 × 2 classroom simulation design with 100 synthetic B2-level writers, this study examines the effects of feedback literacy scripts and a second-review mechanism on writing scores, feedback uptake, revision depth, and delayed performance. It therefore brings written feedback, feedback literacy, and generative AI-supported writing into a shared analytical framework and asks whether structuring feedback processing contributes more to effective revision than adding another feedback source.

## 2 Literature review

### 2.1 Written corrective feedback and revision in second language writing

Research on written feedback in L2 writing has long centered on a fundamental question: does feedback mainly improve the current text, or can it also support longer-term writing development? Truscott’s (1996) critique of grammar correction was important not because it rejected all feedback, but because it encouraged researchers to distinguish between two levels of effect: improvement within a specific task and development of more stable writing ability (Truscott, 1996). Within this debate, Ferris (1999) and Ferris and Roberts (2001) argued that feedback effects depend not only on the feedback itself, but also on its explicitness, task conditions, and the way writers process it (Ferris, 1999; Ferris and Roberts, 2001). Hyland (1998) further showed that different writers may take different revision paths even when responding to the same teacher comment. Later studies by Hyland and Hyland (2006), Ellis (2008), Ferris (2010), and Bitchener and Ferris (2012) gradually shifted attention from feedback types to feedback use, highlighting feedback provision and feedback uptake as two closely related dimensions (Hyland, 1998; Hyland and Hyland, 2006; Ellis, 2008; Ferris, 2010; Bitchener and Ferris, 2012).

Later research refined this perspective by showing that feedback does not influence writing automatically. Before it can shape revision, it must first be noticed, interpreted, and evaluated by the learner. Compared with the simple question of whether a suggestion is followed, factors such as processing depth, value judgment, and retention offer a better explanation of differences in writing performance (Storch and Wigglesworth, 2010). Research on learner engagement likewise suggests that improvement in text quality depends less on the amount of feedback than on whether learners are willing to address higher-order concerns such as argumentation, structure, and content (Han and Hyland, 2015). At the same time, studies of different feedback forms show that their effects are not always stable. Even when grammar-focused feedback improves linguistic accuracy, its contribution to argument quality and genre development remains limited (Shintani et al., 2014). Feedback uptake, therefore, is better understood as a process of attention, judgment, and planning rather than simple compliance.

This issue is especially important in EAP classrooms. EAP writing is not only about correcting language, but also about managing task response, evidence organization, stance development, and genre conventions. For this reason, the effectiveness of feedback should be judged not only by whether a text changes, but also by whether those changes improve the core abilities reflected in assessment criteria. Research on EAP pedagogy and writing assessment has consistently shown that the central challenge lies less in correcting isolated language problems than in helping students translate abstract evaluative standards into workable revision practices (Wingate and Tribble, 2012; Lee, 2017). This provides a direct theoretical basis for examining the role of AI feedback in EAP classrooms.

### 2.2 Automated feedback and generative artificial intelligence in EAP classrooms

The development of automated writing evaluation systems moved feedback research from teacher-written comments toward algorithm-based responses. Warschauer and Grimes (2008) argued that the value of automated writing evaluation lies not in replacing teachers, but in changing the accessibility, frequency, and timing of feedback. Shermis and Burstein (2013) and Cotos (2014) further showed that AWE systems can provide relatively stable feedback on genre, structure, and local language issues (Warschauer and Grimes, 2008; Shermis and Burstein, 2013; Cotos, 2014). Wilson and Czik (2016) found that their effects on student motivation, classroom feedback practices, and text quality depend less on technical sophistication than on how feedback is interpreted and used in instruction. Automated feedback, therefore, should be seen not only as a technological tool, but also as an instructional element shaped by classroom design (Wilson and Czik, 2016).

As research moved closer to the era of generative AI, scholars became increasingly concerned with how learners trust, understand, and use machine feedback. Existing studies show that students do not passively accept automated suggestions, but evaluate them in relation to credibility and usefulness. They also show that the main difficulties of AWE integration are not only technical, but pedagogical, especially when teacher guidance, task integration, and evaluative criteria remain unclear (Ranalli, 2021).

Generative AI has made these issues more visible. Existing studies suggest that large language models can provide feedback that is fast, coherent, and interactive, with clear potential for writing support and classroom use (Dai et al., 2023). At the same time, they may also blur the boundaries of assessment and authorial responsibility. More importantly, greater feedback availability does not automatically lead to better learning. As AI-generated suggestions become more abundant, learners may face greater demands in filtering, comparing, and prioritizing them (Afifi et al., 2023).

Generative AI research and traditional AWE research therefore point to the same classroom issue: machine feedback becomes a meaningful learning resource only when it is embedded in a clear pedagogical structure. This is especially important in EAP instruction, where effective writing development depends not only on receiving feedback, but also on making independent judgments about higher-order revision.

### 2.3 Feedback literacy and its analytical value

If the two strands of literature reviewed above explain why feedback matters and why AI can provide it at scale, feedback literacy research addresses a more central question: how students turn feedback into learning. When Carless and Boud (2018) introduced the concept of student feedback literacy, they focused not on general attitudes, but on learners’ ability to understand feedback, make judgments, regulate emotions, and take action (Carless and Boud, 2018). Winstone et al. (2017) further described feedback use as a series of actionable processes, which is consistent with the emphasis on uptake in L2 writing research (Winstone et al., 2017). In EAP classrooms, this means that feedback is effective not simply because it is given, but because students can decide what should be prioritized, what criteria should guide their choices, and how suggestions can be applied without weakening the communicative purpose of the text.

Recent studies have extended this perspective to L2 writing and generative AI contexts. Existing research generally suggests that feedback is more likely to support motivation, engagement, and revision when learners are given clear standards, workable steps, and opportunities to evaluate the value of comments they receive (Yu et al., 2020; Saeed and Alharbi, 2023; Rad and Mirzaei, 2024). More recent work has begun to frame this issue as AI feedback literacy, emphasizing students’ ability to understand, assess, and anticipate the value of AI-generated feedback in writing contexts (Liu and Deris, 2025). However, current research still focuses more on perceptions, definitions, and preliminary interventions than on how feedback literacy operates in actual revision processes. In particular, the links among feedback scripts, uptake behavior, revision depth, and writing performance remain insufficiently examined. Feedback literacy, therefore, is becoming an important concept for explaining how AI feedback works in classroom settings, but its specific role in shaping revision still requires closer analysis.

### 2.4 Theoretical framework and hypotheses

The preceding literature review suggests that EAP writing revision in generative AI-supported contexts can be understood as a process unfolding through feedback input, feedback processing, and textual restructuring. AI can generate comments and suggestions rapidly, but whether these suggestions are translated into effective revision does not depend simply on their presence. It depends on how learners interpret the suggestions, judge their value, and enact them in concrete revision decisions. In the context examined in this study, the primary AI feedback channel provides the initial suggestions, the feedback literacy script guides learners in processing those suggestions, and the second-review mechanism alters the structure of the feedback sources available to learners. Writing outcomes emerge from this sequence of processing and revision activities rather than from any simple increase in the amount of feedback.

The feedback literacy script operates mainly at the stage of feedback processing. It provides learners with a clearer sequence for judgment, allowing revision priorities to be organized around task requirements, argumentative focus, and evaluative criteria. The concern of the present study is not how many suggestions learners adopt, but whether their limited revision resources are directed toward the aspects that matter most. In EAP writing, these aspects primarily include task response, textual organization, and the use of evidence. By contrast, although the second-review mechanism increases the amount of information available, it also raises the difficulty of comparison, filtering, and selection. Additional feedback, therefore, may not be converted into better writing performance in any stable way.

Accordingly, the present study centers its theoretical analysis on four consecutive components: AI feedback provision, feedback processing, revision depth, and writing outcomes. AI feedback provision constitutes the starting point of revision. Feedback processing reflects how learners identify, evaluate, and select suggestions. Revision depth indicates the level at which those suggestions are ultimately enacted. Writing outcomes capture the differences in performance after revision. Among these variables, argument-level revision deserves particular attention because it involves adjustments to textual structure, argumentative development, and evidence deployment, and is therefore more closely tied to the points at which EAP writing quality actually changes.

On this basis, the study proposes four hypotheses:

- H1: Feedback literacy scripts will improve immediate revision gains, and this improvement will be concentrated mainly in higher-order dimensions such as task response, organization, and evidence.
- H2: Compared with the initial revision task, the effect of the feedback literacy script will be weaker in the transfer task.
- H3: The second-review mechanism will not produce a stable improvement in writing outcomes.
- H4: Compared with feedback uptake rate, the proportion of argument-level revision will have stronger explanatory power for changes in writing scores.

## 3 Methodology

### 3.1 Research design

This study adopted a classroom scenario simulation design to examine the mechanism through which feedback functions in EAP writing. The research subjects were B2-level large language model-based writer agents generated under a unified rule set rather than real students. This design was chosen mainly to control for the influence of language proficiency differences on the results and to concentrate the analysis on two treatment factors: the feedback literacy script and the second-review mechanism. The question addressed in this study was whether, under the same initial AI feedback condition, these two mechanisms would affect how writers absorbed feedback and how they revised their texts. For this purpose, 100 synthetic writers were randomly assigned to a 2 × 2 experimental design. The two independent variables were whether a feedback literacy script was introduced and whether an AI second reviewer was provided. As shown in Table 1, the treatment combinations yielded four experimental groups: G1 served as the control group, G2 received only the script treatment, G3 received only the second-review treatment, and G4 received both treatments.

**Table 1:** Experimental design and task structure.

Condition	No second reviewer	With second reviewer
No script	G1 (*n* = 40)	G3 (*n* = 10)
With script	G2 (*n* = 40)	G4 (*n* = 10)

### 3.2 Task procedure

To facilitate comparison of revision performance across different treatment conditions, the task sequence for the synthetic writers was standardized. Each writer first completed the baseline writing task in Task 1 and received the same initial AI feedback. They then revised Task 1 under their assigned condition, namely with or without the script and with or without the second-review mechanism. After that, the writers proceeded to Task 2, where they completed a new round of writing and revision under a changed topic but the same genre, so that the study could observe whether the feedback-use pattern developed in the previous task would carry over to the next round of writing. Task 3 was a delayed writing task intended to examine whether earlier revision experience would continue to influence writing performance after an interval. All three tasks were situated in an EAP argumentative writing context. Because the primary AI feedback was kept the same across groups, any subsequent differences mainly reflect variation in feedback use under different treatment conditions rather than differences in the feedback content itself.

The “participants” in this study were B2-level writer agents generated through a unified prompt protocol rather than real students. This design was adopted mainly to minimize differences in language proficiency and to concentrate the analysis on the roles of the feedback literacy script and the second-review mechanism in the revision process. During generation, a small number of individual characteristics related to feedback use were retained, including prior AI experience, risk aversion, metacognitive awareness, and typical revision style. However, the range of variation in these characteristics was controlled, and they were not analyzed as separate sources of heterogeneity in the present study. Because the study employed a simulation-based design, it did not involve the recruitment of real human participants or classroom intervention and therefore did not require ethics approval. Table 2 summarizes the main prompt structures and their purposes.

**Table 2:** Main prompt structures.

Stage	Prompt name	Purpose	Core prompt content
Writer generation	Writer initialization prompt	Generate B2-level writer agents with a standardized proficiency profile	“You are a B2-level EAP student writer… Your English proficiency should remain within a narrow B2 range… You may differ slightly in prior AI experience, risk attitude, metacognitive awareness, and revision style, but do not display extreme variation…”
Task 1 baseline writing	Task 1 drafting prompt	Generate the first-round argumentative essay draft	“Write an EAP argumentative essay of approximately … words on the following topic: … Maintain a B2-level writing profile… Do not revise while drafting…”
Primary AI feedback	Primary feedback prompt	Generate a common-source feedback set for all conditions	“Read the essay and provide feedback on task response, organization, evidence, and language… List actionable suggestions… Do not rewrite the whole essay…”
Script processing	FRAC script prompt	Guide writers to process feedback through the script	“Before revising, examine the feedback in the following order: (1) identify the comments most relevant to task requirements; (2) decide which comments affect argument, organization, and evidence; (3) prioritize changes; (4) explain briefly why each selected change matters…”
No-script processing	No-script prompt	Provide no additional structured guidance	“You have received feedback on your draft. Revise the essay as you think appropriate…”
Second review	Second-review prompt	Provide an additional feedback source	“A second reviewer now comments on the same essay… Provide additional comments on strengths, weaknesses, and revision priorities… Avoid repeating all earlier comments unless necessary…”
Task 1 revision	Task 1 revision prompt	Complete the first-round revision under the assigned condition	“Revise your Task 1 essay based on the available feedback under your assigned condition… Keep the original task and genre unchanged…”
Task 2 transfer writing	Task 2 drafting prompt	Test whether the revision approach transfers to a new task	“Write a new argumentative essay on a different topic but in the same genre… Maintain the same writer profile…”
Task 2 revision	Task 2 revision prompt	Conduct feedback processing and revision again on the new topic	“Revise the Task 2 draft using the same condition-specific procedure as assigned previously…”
Task 3 delayed performance	Delayed writing prompt	Observe delayed retention	“Complete the delayed writing task independently… Produce a text under the same B2-level profile…”
Scoring	Scoring prompt/human rubric	Score the overall performance and the four analytic dimensions	“Evaluate the essay on task response, organization, evidence, and language using a 0–100 scale…”/“Blind-score the essay according to a unified rating rubric, …”
Coding	Uptake/depth coding prompt	Code feedback uptake and revision depth	“Code each revision unit as effective uptake/mis-uptake/not adopted… Code revision depth as surface/sentence/paragraph/argument…”

### 3.3 Model specification

The outcome variables used in this study comprised three components: writing quality, feedback uptake, and revision depth. Writing quality was measured by the overall score, total_score, and four analytic dimensions, namely task_response, organization, evidence, and language. All texts were independently scored by two blind raters on a scale from 0 to 100. Feedback uptake was represented by effective_rate, mis_uptake_rate, and not_adopted_rate, which captured the proportions of feedback that was effectively adopted, mis-adopted, or not adopted. Revision depth was coded on a four-level scale, including surface, sentence, paragraph, and argument, corresponding to different levels of revision ranging from surface substitution to argument restructuring. Among these indicators, argument_prop was used to represent the proportion of argument-level revision.

The core treatment variables were operationalized as three indicator variables: feedback literacy script use, AI second-review mechanism, and task period. These variables are formally defined in Equations 1−3.


$${\text{Script}}_{i}=\{\begin{array}{ll} 1, & \begin{array}{l} \text{writeriused the FRAC}\;\\ \text{feedback literacy script} \end{array}\\ 0, & \text{otherwise} \end{array}\;\;\;\;$$ (1)

$${\text{Second}}_{i}=\{\begin{array}{ll} 1, & \begin{array}{l} \text{writerireceived the second}-\\ \text{review mechanism} \end{array}\\ 0, & \text{otherwise} \end{array}\;\;\;\;$$ (2)

$$\text{Task}2_{t}=\{\begin{array}{ll} 1, & \text{the observation comes from Task}\;2\\ 0, & \text{the observation comes from Task}\;1 \end{array}$$ (3)

The control variables mainly included draft score and baseline writing ability. These two control variables are defined in Equations 4 and 5.


$${\text{DraftScore}}_{\mathrm{it}}=\text{the draft score of writerion taskt}$$ (4)

$${\text{Baseline}}_{i}=\text{baseline writing score of writeri}$$ (5)

During the immediate revision stage, the first outcome of interest was revision gain. Revision gain is defined in Equation 6.


$${\text{Gain}}_{\mathrm{it}}={\text{RevisedScore}}_{\mathrm{it}}-{\text{DraftScore}}_{\mathrm{it}}$$ (6)

The model specification is presented in Equation 7:


$$\begin{array}{l} {\text{Gain}}_{\mathrm{it}}=\beta_{0}+\beta_{1}{\text{Script}}_{i}+\beta_{2}{\text{Second}}_{i}+\beta_{3}\text{Task}2_{t}+\\ \;\beta_{4}({\text{Script}}_{i}\times {\text{Second}}_{i})+\beta_{5}({\text{Script}}_{i}\times \text{Task}2_{t})+\\ \;\beta_{6}({\text{Second}}_{i}\times \text{Task}2_{t})+\varepsilon_{\mathrm{it}} \end{array}$$ (7)

In this model, $\beta_{0}$ is the intercept and represents the expected revision gain for the reference condition, that is, writers in the no-script, no-second-review condition in Task 1. ${\text{Script}}_{i}$ is a binary indicator coded 1 if writer i received the feedback literacy script and 0 otherwise. ${\text{Second}}_{i}$ is a binary indicator coded 1 if writer i received the second-review mechanism and 0 otherwise. $\text{Task}2_{t}$ is a task-stage dummy coded 1 for Task 2 and 0 for Task 1. Thus, $\beta_{1}$ represents the average effect of the script in Task 1, $\beta_{2}$ represents the average effect of the second-review mechanism in Task 1, and $\beta_{3}$ captures the overall change from Task 1 to Task 2 for the reference group. The interaction term $\beta_{4}({\text{Script}}_{i}\times {\text{Second}}_{i})$ tests whether the two treatments jointly produce an additional effect beyond their separate contributions. The term $\beta_{5}({\text{Script}}_{i}\times \text{Task}2_{t})$ examines whether the script effect changes in the transfer task, and $\beta_{6}({\text{Second}}_{i}\times \text{Task}2_{t})$ examines whether the effect of the second-review mechanism changes from Task 1 to Task 2. Finally, $\varepsilon_{\mathrm{it}}$ denotes the error term, capturing unexplained variation in revision gain across writers and tasks.

To further control for differences in initial draft quality, the study also estimated a revised-score model, as specified in Equation 8:


$$\begin{array}{l} {\text{RevisedScore}}_{\mathrm{it}}=\alpha_{0}+\alpha_{1}{\text{DraftScore}}_{\mathrm{it}}+\alpha_{2}{\text{Script}}_{i}+\\ \;\alpha_{3}{\text{Second}}_{i}+\alpha_{4}\text{Task}2_{t}+\\ \;\alpha_{5}({\text{Script}}_{i}\times \text{Task}2_{t})+u_{\mathrm{it}}\;\; \end{array}$$ (8)

In this model, $\alpha_{0}$ is the intercept, and ${\text{DraftScore}}_{\mathrm{it}}$ is included as a control variable to account for the influence of initial text quality on the revised score. Accordingly, $\alpha_{1}$ captures the extent to which stronger drafts are associated with higher post-revision scores. $\alpha_{2}$ and $\alpha_{3}$ represent the effects of the script and the second-review mechanism, respectively, after draft quality has been taken into account. $\alpha_{4}$ reflects the overall difference between Task 1 and Task 2, and $\alpha_{5}$ captures whether the effect of the script changes in the transfer task. The disturbance term $u_{\mathrm{it}}$ represents residual variation in revised scores not explained by the observed predictors.

In the delayed stage, retention was assessed through performance on Task 3, using the delayed retention model specified in Equation 9:


$$\begin{array}{l} {\text{DelayScore}}_{i}=\gamma_{0}+\gamma_{1}{\text{Script}}_{i}+\gamma_{2}{\text{Second}}_{i}+\\ \;\gamma_{3}({\text{Script}}_{i}\times {\text{Second}}_{i})+\gamma_{4}{\text{Baseline}}_{i}+\eta_{i} \end{array}$$ (9)

where ${\text{DelayScore}}_{i}$ denotes the delayed writing score of writer i in Task 3, and ${\text{Baseline}}_{i}$ denotes the baseline writing ability score of writer i before the experiment. Because each writer contributed only one observation at this stage, no task dummy variable was included. In this model, $\gamma_{0}$ is the intercept, $\gamma_{1}$ and $\gamma_{2}$ represent the effects of the script and the second-review mechanism on delayed performance, and $\gamma_{3}$ tests whether the combined presence of the two treatments is associated with an additional delayed effect. $\gamma_{4}$ controls for prior writing ability so that delayed performance can be interpreted relative to each writer’s starting level. The error term $\eta_{i}$ captures the remaining unexplained variation in delayed scores across writers.

In addition to the score models, the study also treated feedback uptake indicators and revision depth indicators as mechanism-oriented outcome variables in order to explain how treatment effects emerged. The former addressed whether feedback was correctly absorbed, whereas the latter captured whether revision actually moved toward higher-level structural reworking. Within this set of indicators, argument_prop was especially important because it reflects not local textual adjustment, but deeper changes at the level of argument structure.

Given the unequal group sizes, especially the comparatively smaller second-review conditions, all models were estimated using HC3 robust standard errors to reduce reliance on the homoskedasticity assumption. Because treatment assignment was exogenous by design, conventional endogeneity was not the main identification concern. More relevant issues were estimation volatility under uneven cell sizes, possible baseline imbalance, and potential bias in scoring and coding. To address these concerns, four design diagnostics were included. First, a baseline balance test was conducted to compare initial writing ability between the script and non-script groups. Second, exact permutation tests were used for the script effect in Task 1 and the retention effect in Task 3. Third, leave-one-out sensitivity analysis was conducted to assess whether the results were overly influenced by individual writers. Fourth, inter-rater ICC values and inter-coder agreement indices were reported to support the reliability of scoring and coding.

### 3.4 Diagnostic checks

Given the unequal group sizes, with the second-review conditions in particular having relatively small samples, this study reports diagnostic and robustness checks separately. All models were estimated using HC3 robust standard errors. The main issues requiring evaluation in this study were whether the estimates remained stable under sample imbalance, whether there were initial between-group differences that might affect comparison, and whether scoring and coding introduced potential sources of error. On this basis, four checks were conducted: initial writing ability was compared between the script and no-script groups to assess baseline balance; exact permutation tests were performed for the script effect in Task 1 and the retention effect in Task 3; a leave-one-out procedure was used to examine the sensitivity of the results to individual writers; and inter-rater ICC values together with inter-coder agreement indices were reported.

## 4 Results

### 4.1 Descriptive results and baseline check

The descriptive statistics and baseline checks indicate that the groups differed little in their initial writing ability. When combined by script condition, the scripted group showed a slightly lower baseline mean than the non-scripted group, but the Welch test did not indicate a significant difference. Table 3 also shows that G1 and G2 had larger sample sizes than G3 and G4. However, the total sample sizes of the scripted and non-scripted conditions remained broadly comparable. Therefore, overall comparisons centered on the script condition are relatively stable, whereas descriptive differences involving G3 and G4 should be treated more cautiously and mainly as reference points. Table 3 presents the group composition and baseline profile of each group.

**Table 3:** Group profile and baseline balance.

Group	N	Script	Second reviewer	CEFR	Baseline writing mean
G1	40	No	No	B2	65.0
G2	40	Yes	No	B2	65.0
G3	10	No	Yes	B2	65.0
G4	10	Yes	Yes	B2	63.0

**Figure 1:**
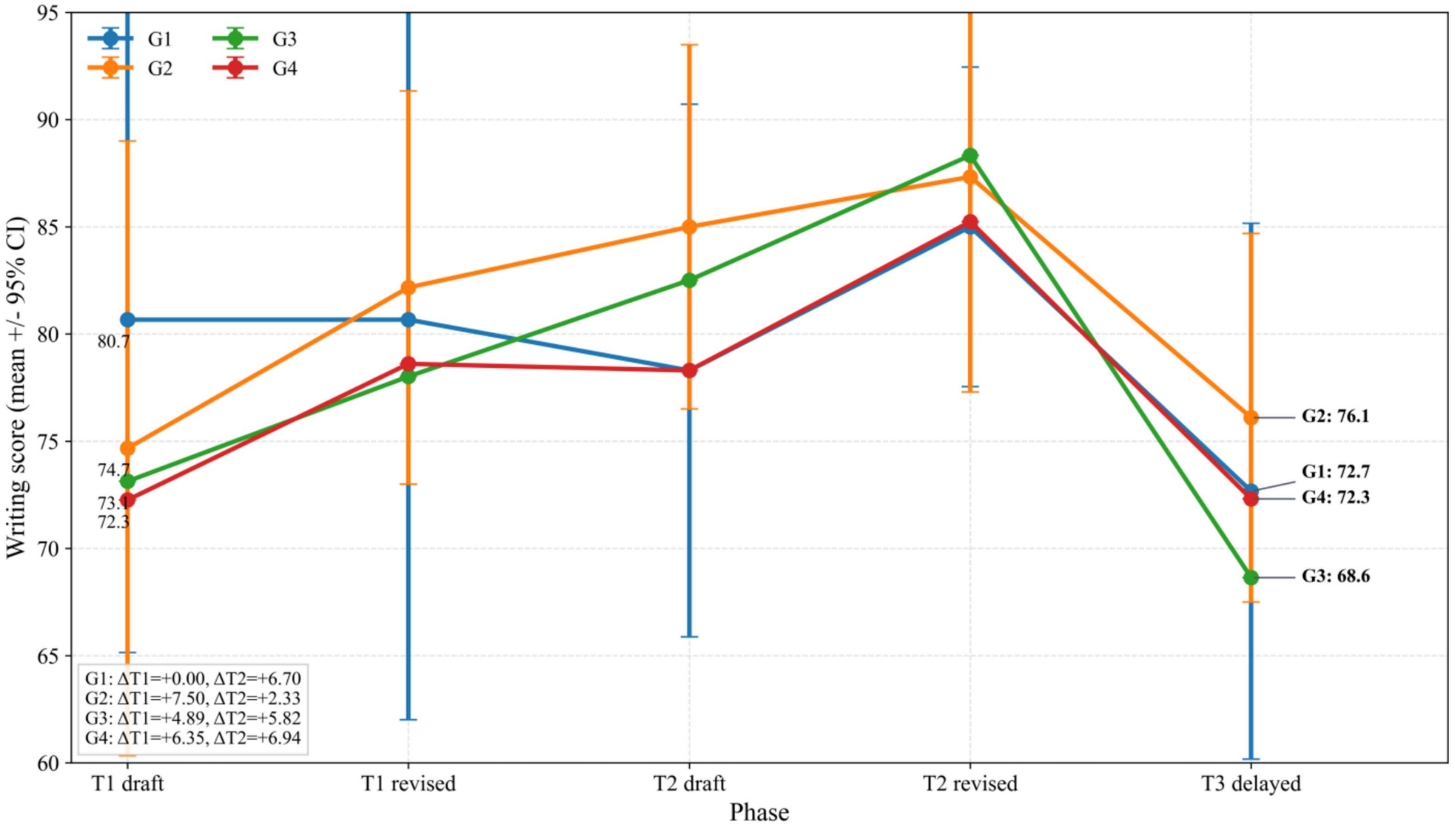
Writing-score trajectories across phases.

In terms of group mean trajectories, the most pronounced improvement associated with the script treatment appeared in Task 1. G2 increased from 74.67 to 82.17, yielding a mean gain of 7.50 points, whereas the corresponding control group, G1, remained largely stable in Task 1. In Task 2, however, G1 rose from 78.00 to 85.00, a gain of 7.00 points, while G2 increased from 85.00 to 87.33, a gain of 2.33 points. Considered together with the initial draft levels of the two groups, this pattern can be interpreted as a natural shift in growth potential and strategy use across task stages. On the one hand, G2 had already reached a relatively high level at the draft stage of Task 2, indicating substantial gains from the earlier phase. On the other hand, the transfer task required writers to apply revision awareness developed in the previous round to a new topic more independently, making it a more revealing indicator of strategy internalization. In the delayed stage, the scripted group obtained a mean score of 76.00, higher than the 72.67 observed for the non-scripted group, suggesting that the script treatment continued to show a positive signal at the retention stage. Table 4 and Figure 1 provide a fuller presentation of these trends.

**Table 4:** Descriptive statistics of writing scores by group and phase.

Group	T1 draft	T1 revised	T2 draft	T2 revised	T3 delayed
G1	80.67 ± 6.25	80.67 ± 7.51	78.30 ± 5.00	85.00 ± 3.00	72.67 ± 5.03
G2	74.67 ± 5.77	82.17 ± 3.69	85.00 ± 3.42	87.33 ± 4.04	76.10 ± 3.46
G3	73.13 ± 4.82	78.02 ± 4.15	82.51 ± 3.96	88.33 ± 3.42	68.64 ± 4.27
G4	72.26 ± 5.11	78.61 ± 4.36	78.30 ± 4.28	85.24 ± 3.75	72.31 ± 4.03

Values are reported as means ± standard deviations.

### 4.2 Main regression results

The regression results in Table 5 are broadly consistent with the descriptive patterns reported above. Model 1 shows that, in the gain model pooling Task 1 and Task 2, the coefficient for the script variable was 5.85 (SE = 3.81), and the Task 2 dummy was also positive, indicating that revision gains could still be observed in the transfer task. The coefficient for Script × Task 2 was −8.88, suggesting that the script effect was concentrated mainly in the stage when writers first encountered AI feedback and became weaker in Task 2. After controlling for draft scores, Model 2 yielded a script coefficient of 4.26 and a DRAFT coefficient of 0.68, with the latter reaching statistical significance. This indicates that revised-draft scores were associated with both initial draft quality and the script treatment. Across these main models, the direction of the script coefficient remained consistently positive, whereas the second-review estimates were accompanied by much larger standard errors and were therefore less precise. Given the unequal cell sizes, especially the relatively small second-review groups, these results should be interpreted together with the HC3 robust standard errors.

**Table 5:** Main model estimates for revision gain and delayed retention.

Term	Model 1: pooled gain	Model 2: revised score	Model 3: Task 1 gain	Model 4: delayed score
Script	5.85 (3.81), *p* = 0.125	4.26 (3.10), *p* = 0.170	7.09 (4.33), *p* = 0.102	3.33 (4.32), *p* = 0.440
Second reviewer	1.71 (11.50), *p* = 0.882	0.55 (2.01), *p* = 0.786	4.47 (6.83), *p* = 0.513	−4.67 (12.17), *p* = 0.701
Script × Second reviewer	−0.42 (13.37), *p* = 0.975	—	−6.62 (16.13), *p* = 0.682	2.90 (34.30), *p* = 0.933
Task 2	5.35 (4.40), *p* = 0.224	5.50 (2.63), *p* = 0.037	—	—
Script × Task 2	−8.88 (7.47), *p* = 0.235	−6.05 (4.60), *p* = 0.188	—	—
Draft/Baseline	—	0.68 (0.20), *p* = 0.001	−0.07 (0.42), *p* = 0.869	1.12 (0.06), *p* < 0.001
$R^{2}$	0.449	0.744	0.861	0.414
N	200	200	100	100

When examined by stage, the Task 1-only model more clearly captures the role of the script in the first revision round. In Model 3, the coefficient of the script on Task 1 gain was 7.09. Correspondingly, the mean gain difference between the scripted and non-scripted groups in Task 1 was 5.75 points, and the exact permutation test yielded *p* = 0.0286. Taken together, these two results indicate that the script already showed a fairly clear positive contribution when writers first processed AI feedback. By contrast, the second-review mechanism did not display a similarly stable pattern. In Model 4, the coefficient of the script on delayed Task 3 scores was 3.33, and the scripted group also obtained a higher mean score than the non-scripted group. However, the precision of the estimate at this stage was limited, and the exact permutation test did not provide sufficiently strong support. The delayed-stage results are therefore better treated as directional evidence rather than as a stable retention effect. Overall, the script effect was concentrated mainly in the initial revision stage, whereas the second-review mechanism did not show a stable contribution under the present sample configuration.

### 4.3 Heterogeneity and underlying mechanisms

If only the total score is considered, the script effect can easily be interpreted as a generalized overall improvement. The dimension-specific results, however, show that the advantage of the script treatment had a much clearer locus of effect. Table 6 shows that, in Task 1, the script significantly improved task response β = 1.50, *p* = 0.0285, organization β = 1.25, *p* = 0.0083, and evidence β = 2.25, *p* = 0.0088, while also showing a positive influence on the language dimension. This indicates that the main value of the script did not lie merely in surface-level polishing, but in directing writers’ attention more effectively toward the higher-order dimensions that are more central to EAP writing. At the same time, the interaction between Script and Task 2 was negative across the first three dimensions, suggesting that the advantage established during the initial revision stage took on a different form in the transfer task. Rather than relying simply on an explicit script, writers gradually began to demonstrate a greater capacity to allocate higher-order revision resources more independently in the new task.

**Table 6:** Dimension-specific heterogeneity of script effects.

Dimension	T1 no script	T1 script	T2 no script	T2 script	$\beta_{\text{script}}$	p	$\beta_{\text{script}\times T2}$	p
Task response	0.13	1.63	1.75	0.75	1.50	0.029	−2.50	0.005
Organization	0.38	1.63	1.63	0.75	1.25	0.008	−2.13	0.043
Evidence	0.63	2.88	2.25	1.50	2.25	0.009	−3.00	0.026
Language	0.13	0.88	1.00	0.50	0.75	0.301	−1.25	0.295

**Figure 2:**
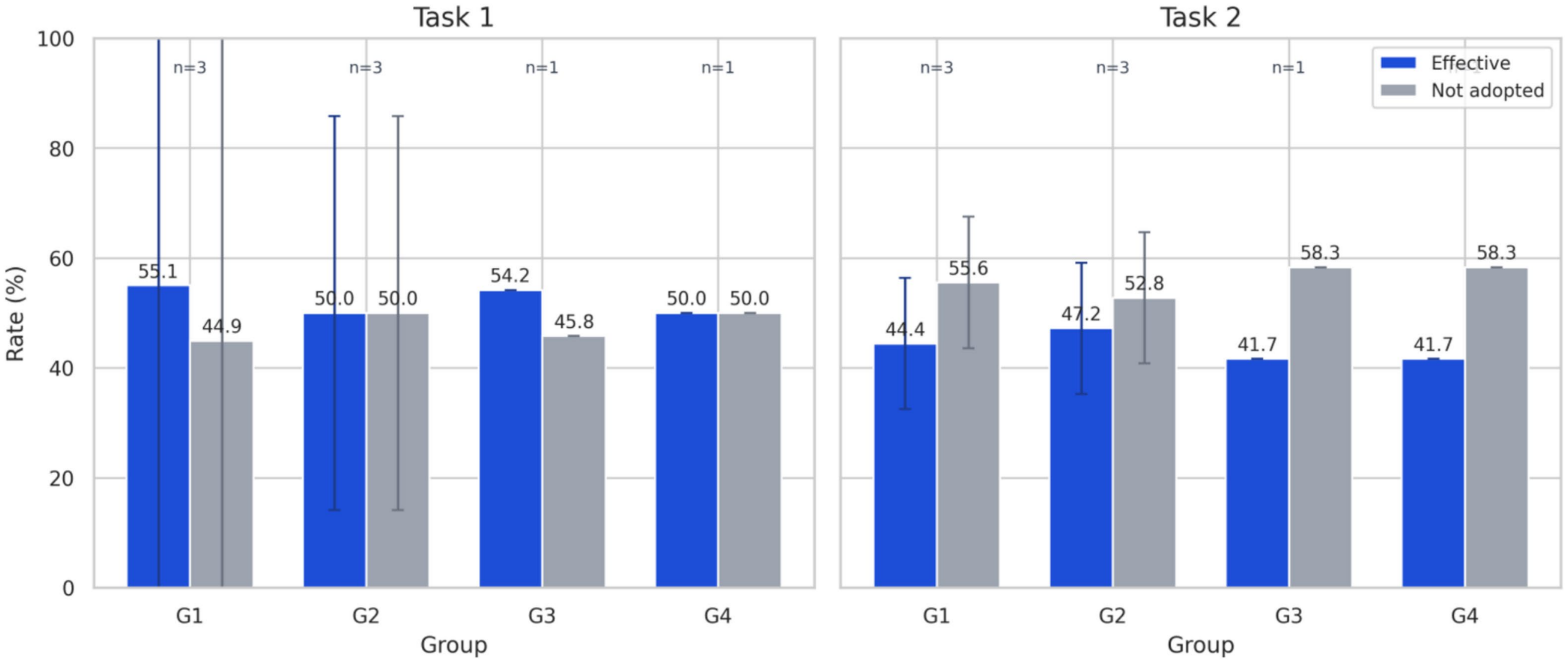
Feedback uptake across groups and tasks.

**Figure 3:**
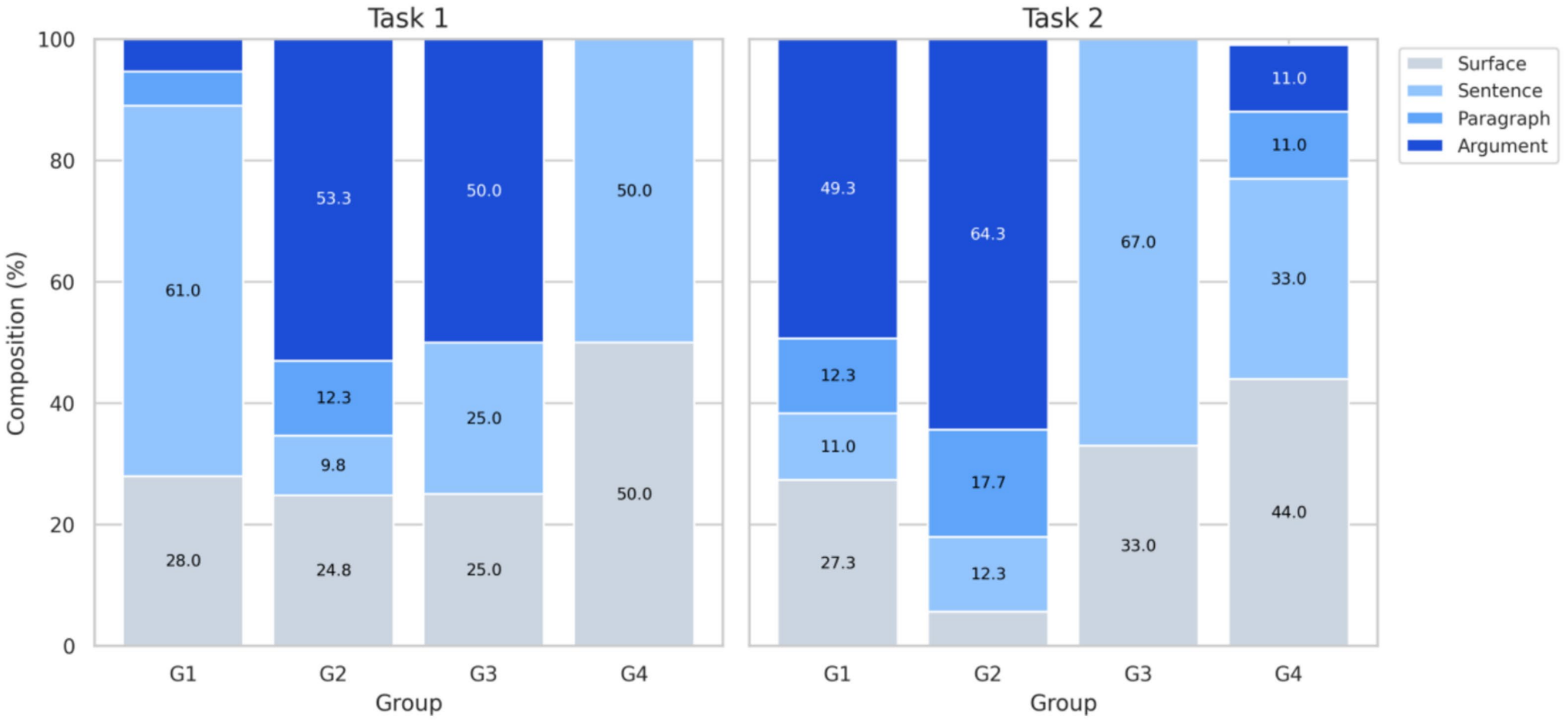
Revision-depth composition by group and task.

**Figure 4:**
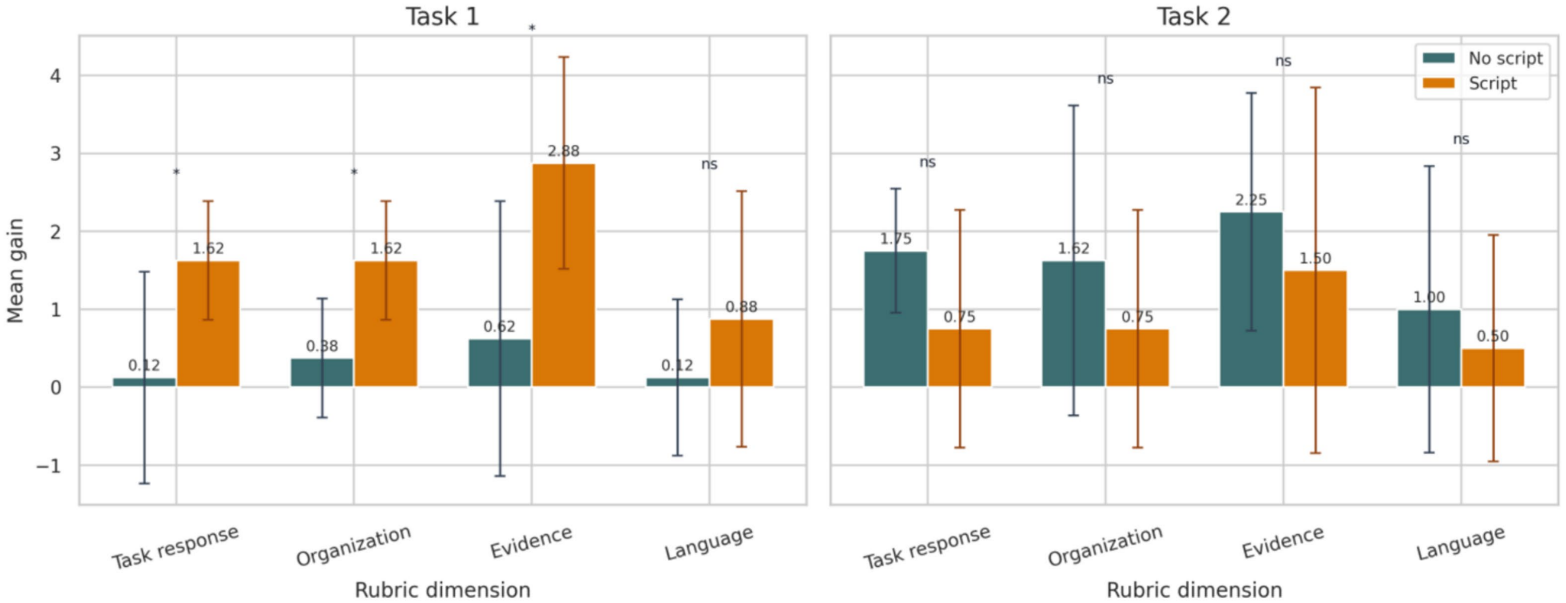
Dimension-specific gains by script condition.

Feedback uptake itself showed another clear pattern. Figure 2 indicates that, across the four groups, the effective uptake rate in Task 1 and Task 2 ranged from about 41.7 to 55.1%, whereas the non-adoption rate ranged from 44.9 to 58.3%. Mis-uptake was close to zero in the present dataset. This suggests that writers across groups were generally able to handle AI feedback in a stable way. More importantly, Figure 3 shows that the scripted groups displayed a more higher-order revision profile. In Task 1, revision in G1 remained mainly at the sentence level, accounting for 61.0%, whereas argument-level revision in G2 reached 53.3%. In Task 2, the proportion of argument-level revision in G2 remained at 64.3%, higher than that of G1 at 49.3% and G4 at 11.0%. This indicates that the value of the script lies not only in whether feedback is adopted, but also in how it is adopted, namely, whether it is turned into higher-level textual restructuring. This interpretation is further supported by Figure 4, which shows that the scripted condition in Task 1 produced larger gains in higher-order rubric dimensions, especially task response, organization, and evidence, whereas the non-script condition showed only limited improvement in these areas. In other words, the script appears to redirect revision effort toward dimensions more closely tied to discourse-level quality rather than merely local linguistic adjustment.

Task 1 provides a clearer view of the underlying mechanism. Argument-level revision was positively correlated with score gain (r = 0.755, *p* = 0.0304), whereas surface-level revision was not. This suggests that the script improved writing performance by shifting revision toward higher-order textual restructuring, not by simply increasing the amount of feedback uptake. The key difference, therefore, lies in revision quality rather than feedback quantity.

### 4.4 Robustness checks

Table 7 reports the robustness checks. The ICC(2,1) for the overall score was 0.9907, and the mean absolute difference between the two raters was 0.2821 points. Cohen’s *κ* for feedback-uptake coding was 0.992. For revision depth, the correlation between the two coders in the proportion of argument-level revision was 0.8219. These results indicate a high level of consistency in both scoring and coding.

**Table 7:** Reliability and robustness diagnostics.

Metric	Value	Interpretation
Score ICC(2,1)	0.9907	Excellent rater agreement
Mean absolute score difference	0.2821	Very small scoring gap
Uptake Cohen’s κ	0.9920	Near-perfect coding agreement
Correlation of argument-level depth share	0.8219	High coder consistency
Exact permutation p for Task 1 script effect	0.0286	Design-based support for immediate effect
Exact permutation p for Task 3 script effect	0.4000	No stable retention effect
Leave-one-out range of Task 1 script effect	4.75 to 7.00	Direction remains positive
Leave-one-out range of Task 3 script effect	2.33 to 5.67	Positive but unstable
Correlation between Task 1 argument share and gain	0.7550	Mechanism-consistent association

The leave-one-out analysis showed that the script effect ranged from 4.75 to 7.00 points in Task 1 and from 2.33 to 5.67 points in Task 3, remaining positive in every iteration for both tasks. The exact permutation tests further distinguished the two stages: the result for Task 1 was *p* = 0.0286, whereas that for Task 3 was *p* = 0.4000. On this basis, the script effect in Task 1 can be regarded as relatively robust, whereas the pattern in Task 3 is better interpreted as directional evidence.

## 5 Discussion

This study suggests that the effectiveness of revision in generative AI-supported EAP writing depends less on the amount of feedback available than on how learners process that feedback. Across the descriptive results, regression models, and design-based tests, the feedback literacy script showed its clearest effect in Task 1, where writers first encountered and acted on AI-generated comments. This pattern is consistent with feedback literacy research, which has emphasized that feedback becomes pedagogically valuable only when learners can interpret it, evaluate its relevance, and convert it into revision decisions (Winstone et al., 2017; Carless and Boud, 2018). In AI-supported contexts, this issue becomes more salient because the rapid production of multiple suggestions may increase access to feedback without necessarily improving learners’ capacity to judge and prioritize it (Liu and Deris, 2025).

A more important result is that the script changed the level at which revision was carried out. In Task 1, the scripted group did not mainly improve by adopting more feedback items. Instead, the difference appeared in the proportion of revision directed to argument, organization, and evidence. The positive association between argument-level revision and score gain further supports this interpretation. This finding matters for EAP writing because its assessment criteria extend beyond linguistic accuracy to include task fulfillment, discourse organization, and evidential support (Lee, 2017). In this sense, the script did not function as a device for increasing compliance with feedback. Its role was to redirect limited revision effort toward dimensions that more directly determine text quality. This reading also aligns with prior work showing that the effect of feedback depends not only on uptake itself, but on the depth and focus of learner engagement with it (Rad and Mirzaei, 2024).

The weaker script effect in Task 2 deserves more careful interpretation. One explanation is that the script operated most strongly when writers first needed external support to organize AI feedback. In Task 1, the script provided an explicit sequence for sorting comments, identifying priorities, and linking suggestions to evaluative criteria. By the time writers moved to Task 2, part of this organizing function may already have been internalized. Under that condition, the role of the script becomes less visible in coefficient size, not necessarily because it ceased to matter, but because the transfer task no longer tests initial support in the same way. A second explanation is that Task 2 required writers to apply earlier revision awareness to a new topic more independently. What was being assessed was therefore not only whether writers still had access to the script, but whether they could carry forward the evaluative logic embedded in it. From this perspective, the reduced Task 2 effect may reflect a shift from externally guided revision to partially internalized revision behavior. For real classrooms, this implies that scripting may be especially useful at the stage when students first learn how to process AI feedback, while later tasks may require less explicit scripting and more opportunities to practice transfer across prompts and topics.

The absence of a stable benefit from the second-review mechanism also has practical significance. Under the present conditions, adding another feedback source did not produce a reliable improvement in writing outcomes. A likely reason is that multi-source feedback increases informational supply and, at the same time, increases the demands of comparison, filtering, and prioritization. Previous studies on automated and AI-generated feedback have similarly noted that learners may struggle when multiple comments are available but decision criteria remain unclear (Wilson and Czik, 2016; Afifi et al., 2023; Yan and Zhang, 2024). For writers who have not yet established a stable revision procedure, more feedback may widen the range of possible actions without improving the quality of choice. In classroom terms, this suggests that the pedagogical value of AI does not increase linearly with the number of comments or reviewers. Without a structure for evaluating feedback, additional input may diffuse rather than sharpen revision focus.

These findings have implications for both teaching and research. For EAP pedagogy, AI-supported revision should be designed not simply as a feedback-rich environment, but as a process in which learners are helped to distinguish higher-order issues from surface-level ones, prioritize revisions according to task demands, and review whether implemented changes actually strengthen the text. For research, the results indicate that final scores alone do not sufficiently capture how AI-supported revision works. Feedback uptake and revision depth need to be examined together, because they reveal whether performance changes are driven by superficial correction or by more substantive restructuring. At the same time, the present study remains simulation-based. Its value lies in clarifying a plausible mechanism under controlled conditions, not in replacing evidence from authentic classrooms. Future work should therefore test whether the script effect can be reproduced with human writers, whether its influence changes across proficiency levels, and how scripting interacts with more complex feedback ecologies in real EAP instruction.

## 6 Conclusion

This study used a large language model-driven classroom simulation and an exploratory 2 × 2 design to examine the roles of a feedback literacy script and a second-review mechanism in generative AI-supported EAP writing revision. Across three task stages, it analyzed writing performance, feedback uptake, revision depth, and delayed retention.

The results show that the feedback literacy script played a clearer role than the second-review mechanism. Its effect was most visible in the initial revision stage, where it did not mainly increase the amount of feedback uptake, but shifted revision toward higher-order dimensions such as task response, organization, and evidence use. In addition, argument-level revision was more closely associated with score improvement than feedback uptake quantity alone. This suggests that effective AI-supported EAP revision depends less on the number of feedback sources than on whether learners can process and implement feedback through structured decision-making.

By contrast, the second-review mechanism did not show a stable advantage under the present conditions. From a pedagogical perspective, the value of generative AI in EAP writing should therefore be understood less as an expansion of feedback quantity and more as support for organized feedback processing. At the same time, the study remains simulation-based, and the second-review groups were relatively small. The findings should therefore be validated further in authentic EAP classrooms. Overall, the study contributes by highlighting revision depth, especially argument-level revision, as a key process variable and by offering support for feedback scripting as a practicable instructional pathway in AI-assisted EAP writing.

## Data Availability

The raw data supporting the conclusions of this article will be made available by the authors, without undue reservation.
